# Amputation rate of diabetic foot ulcer and associated factors in diabetes mellitus patients admitted to Nekemte referral hospital, western Ethiopia: prospective observational study

**DOI:** 10.1186/s13047-020-00433-9

**Published:** 2020-11-04

**Authors:** Firomsa Bekele, Legese Chelkeba

**Affiliations:** 1 Department of Pharmacy, College of Health Science, Mettu University, Mettu, Ethiopia; 2 School of Pharmacy, College of Health Science, Addis Ababa University, Addis Ababa, Ethiopia

**Keywords:** Diabetes foot ulcer, Diabetes mellitus, Amputation rate, Associated factors, Nekemte referral hospital

## Abstract

**Background:**

Diabetes foot ulcer is a devastating and much-feared complication of diabetes. Diabetes foot ulcerations which developed gangrene can take weeks or months to heal and can sometimes not heal at all so that amputation for non-traumatic causes is a frequent outcome in the diabetic foot. Despite this, there is no finding on predictors of the amputation rate of diabetes foot ulcers in Ethiopia. Hence this study was aimed to identify factors associated with the amputation rate of diabetes foot ulcer patients in Nekemte referral hospital.

**Patients and methods:**

A prospective observational study was conducted among adult diabetes foot ulcer patients admitted to Nekemte referral hospital from March 15 to June 15, 2018. A pus swab was obtained from the ulcers before any ulcer cleaning to conduct gram staining. The primary outcome was the amputation rate. Cox regression analysis was used to estimate the hazard ratios and time from study entry to healing was evaluated as censored event times by Kaplan-Meier curves.

**Result:**

Over the study period, 115 diabetes foot ulcer patients were admitted to the NRH; of these patients, 64(55.65%) were males while the mean age of participants was 44.4 ± 14.7. A total of 34(29.57%) of the diabetes foot ulcer were overweight and 16(13.91%) were obese while the mean ± standard deviation of body mass index (BMI) was 24.94 ± 3.69 kg/m2 and a total of 56(48.69%) diabetic foot ulcer had a diabetic complication.

Of patients with diabetic foot ulcer, 35(30.43%) were undergone lower extremity amputations (LEA). Patients who were prescribed with inappropriate antibiotics were unlikely to heal. A total of 18(46.15%) of the patients who were taken inappropriate antibiotics were healed whereas 21(53.85%) were not healed (*P* = 0.017). Besides, the higher the Wagner grade, the worse the outcome of healing. A total of 19(21.84%) and 16(57.14%) of patients with grade < 4 and grade ≥ 4, respectively, did not heal (*P* = 0.005).

**Conclusion:**

The amputation rate of diabetes foot ulcers was rapid for patients prescribed inappropriate antibiotics and higher grades of the foot ulcer. Therefore, the presence of clinical pharmacists plays a pivotal role to promote the appropriate use of antibiotics and besides the daily care, special attention should be given for patients having an advanced grade of diabetes foot ulcer.

## Background

Diabetes mellitus is a chronic disease that needs long-term medical attention to prevent the development of its complications [1]. The development of diabetes foot ulcers increases a patient’s risk of mortality [2].

Different treatment protocols which include applying vascular intervention, anti-infection treatment, surgical operation, and postoperative wound care have been performed to increase the healing rate of the diabetes foot ulcer. Despite these, the reported diabetic foot ulcer healing rates from multiple series were poor [3].

Amputation of the lower extremities is the commonly occurred outcome for the DFU [4]. Approximately, more than half were progressed to infections that may result in amputations, disability, prolonged hospitalization, and death [5]. Due to prolonged healing time, many patients will need to be hospitalized for treatment [6]. For people with diabetes who have an active ulcer, the final healing rates are 65–75% for those attending a hospital, while around 15–20% of all people with an ulcer undergo amputation, depending on the duration of follow-up [7].

The size of the ulcer was greater in the amputation group compared to healed ulcers which can predict diabetes foot ulcer healing [8]. Despite this, the feet of diabetes patients were ignored by health care providers which could have an economic impact on the patient and health care system as a result of long-term in-hospital treatment and/or amputation [1, 9].

Determinants of ulcer healing in diabetes patients are generally essential in establishing management strategy in addition to their routine application as predictors of the outcome. Therefore, they are useful in the early identification of diabetes patients with high risk for foot ulcers to decrease the risk of amputation [10].

Diabetes mellitus patients whose ulcer progressed to Chronic do not show the well-defined sequence of ulcer healing. An increase in wound size and surface may empirically be determinants of poor healing [2]. In Ethiopia, patient behavior of poor diabetes foot ulcer treatment practice, and the absence of good quality service of diabetes foot ulcer may have led to foot infections which result in limb amputation. Only a few pharmacists were assigned to avoid the inappropriate use of antibiotics in the Nekemte referral hospital by intervening problems at only dispensing levels. Despite this, no study has been conducted on the amputation rate of diabetic foot ulcers in NRH. Therefore, this study was tried to determine the factor that affect the amputation rate of diabetes foot ulcer patients.

## Methods

### Study design, period and area

A prospective observational study was conducted at NRH from March 15 to June 15, 2018 to assess the amputation rate of diabetes foot ulcers. The hospital is found in Nekemte town, which is located 330 km to the west of Addis Ababa, the capital city of Ethiopia. The hospital is a referral hospital and gives health services for more than 10 million people living in western Ethiopia. There were about 2420 diabetic patients who have been following the diabetic clinics annually.

### Study participants and eligibility criteria

Patients ≥18 years who were admitted to the hospital due to no- traumatic chronic diabetes foot ulcer with visible foot lesions were included.

### Study variables and outcomes

The primary outcome was the amputation rate. Independent variables included were sex, age, residence, educational level, marital status, type of diabetes mellitus, antibiotic given, previous history of ulcer, grade of diabetes foot ulcer, and presence of co-morbidity. The Wagner classification of diabetes foot ulcers was used to assess the grades of foot ulcers. The magnitude of foot ulcer was determined by multiplying the largest by the second largest diameter perpendicular to the first [11]. The etiology of diabetes foot infection was identified by using gram stains. Amputation and healing status was measured using a checklist and assessed by close followed of the patient through a telephone interview of the patient/ caregiver/ proxy on a weekly basis. The ulcer of different sites which includes, dorsal/inter-digital toes, plantar fore foot/mid foot/hind foot, plantar toes, dorsal foot, and heel were recorded to identify the location of diabetes foot ulcer.

### Sample size and sampling technique

Single population proportion formula was used to calculate the required sample size by considering the incidence of amputation which is 29% [12]. Accordingly, a sample of 316 was obtained. The expected number of source population in the study period, based on the average number of patients coming to the hospital was 156. Finally, by using correction formula a total of 115 patients were included. Conveniently all patients during the study period full filling the eligibility criteria and willing to respond were included in the final analysis.

### Data collection process and management

Data was collected using data abstraction format which was developed after reviewing different literature and adopting [5, 7–12]. One medical doctor, one nurse and one pharmacist were selected as data collectors. One medical doctor working in a medical ward who had not been involved in data collection was assigned to supervise the collected data. A pus swab was obtained from the ulcers before any ulcer cleaning and avoiding other contamination. The samples were delivered to the laboratory immediately and a thin smear was prepared on grease or oil free slides. Appropriateness of antibiotics was identified based on infectious diseases society of America (IDSA) standard guidelines for diagnosis and treatment of diabetes foot infection [13], which is based on the most likely coverage of antibiotics for treatments of diabetic foot infection for identified gram stain results and their correct dosage regimens. Five percent of the sample was pre-tested to check the acceptability and consistency of the data collection tool 2 weeks before the actual data collection.

### Data processing and analysis

The data was entered into the computer using EPI-manager 4.0.2 and analysis was done using statistical package for social sciences (SPSS) 24. Descriptive data was explained by frequency and percentage. The obtained results were explained by means and standard deviations (SD). Cox regression analysis was used to estimate the hazard ratios and Time from study entry to time to healing was evaluated as censored event times by Kaplan-Meier curves. The variables with a *p*-value of less than 0.05 had a statistically significant association with the healing of diabetic foot ulcers.

### Operational definitions

**Table:** 

**Chronic diabetes foot ulcer:** Is defined as a foot ulcer unable to heal after 4 weeks [14]. **Healed:** The complete closure of the diabetes foot ulcer with normal skin and without, drainage or sinus formation. **Amputation:** Removal of lower extremity limb which includes both below ankle (minor) and below knee (major). **Appropriate antibiotics:** Antibiotics prescribed per the infectious diseases society of America (IDSA) guideline for the diagnosis and treatment of diabetic foot infection recommendation based on gram stains and dosage regimens. **Inappropriate antibiotics:** Antibiotics prescribed inconsistent with the infectious diseases society of America (IDSA) guideline for the diagnosis and treatment of diabetes foot infection recommendation based on gram stains and dosage regimens. **Grades of diabetes foot ulcer:** For the purpose of this study we used the Wagner system for classification of diabetic foot ulcer which uses 6 wound grades (scored 0 to 5) to assess ulcer depth [15]. • Grade 0 diabetes foot ulcer: No ulcer, but the foot is at risk for ulceration • Grade 1 diabetes foot ulcer: Superficial ulceration • Grade 2 diabetes foot ulcer: Ulcer with deep infection, but without involvement of the bone • Grade 3 diabetes foot ulcer: Ulcer with osteomyelitis. • Grade 4 diabetes foot ulcer: Presence of localized gangrene on the foot. • Grade 5 diabetes foot ulcer: Presence of gangrene of the whole foot.	

## Result

### Socio-demographic and clinical characteristics

During the study period, 115 diabetes foot ulcer patients were admitted to the NRH; of these patients, 64(55.65%) were males. A total of 26(22.61%) of them were in the age range of 58–67, and 56(48.69%) of them had hypertension as comorbidity (Table 1). A total of 34(29.57%) of the diabetes foot ulcer were overweight and 16(13.91%) were obese while the mean body mass index (BMI) was 24.94 ± 3.69 kg/m^2^. From the sites of ulcers involved, a total of 67(58.26%) of them were developed over plantar toes/foot whereas, 31(26.96%) of ulcers were located on dorsal/interdigital toes, 9(7.83%) of the diabetes foot ulcers were located in the dorsal foot and 8(6.96%) of the ulcers were developed over heel [16]. The mean fasting blood glucose level among diabetic patients with foot ulcers was 147.93 ± 45.03 mg/dl and a total of 56(48.69%) diabetes foot ulcers had a diabetes complication.

**Table 1:** Demographic and clinical patient characteristics of diabetes foot ulcer patients in Nekemte referral hospital, west Ethiopia, 2018

Variables		Frequency (n)	Percent (%)
Gender	Male	64	55.65
	Female	51	44.35
Age (years)	18–27	16	13.91
	28–37	14	12.17
	38–47	15	13.04
	48–57	24	20.87
	58–67	26	22.61
	68–77	20	17.39
Types of DM	Type 1	54	46.96
	Type 2	61	53.04
Residence	Urban	58	50.43
	Rural	57	49.57
Educational level	Illiterate	24	20.87
	Primary school	29	25.22
	Secondary school	22	19.13
	Above Secondary school	40	34.78
Marital status	Married	80	69.57
	Single	21	18.26
	Widow	8	6.96
	Divorced	6	5.22
Types of co-morbidity	Hypertension	56	48.69
	Dyslipidemia	40	34.78
	Coronary heart disease/ischemic heart disease	41	35.65
	Peripheral vascular disease	42	36.65
Wagner’s grade	Grade < 4	83	72.17
	Grade ≥ 4	32	27.83
Antibiotics given	Appropriate	38	49.35
	Inappropriate	39	50.65

### Healing time of diabetes foot ulcer and associated factors

Over the study period, a total 35(30.43%) patients were undergone lower extremity amputations (LEA) and 80(69.57%) were healed. Regarding the rate of wound healing (in cm^2^/week): a total of 20(17.39%) were healed < 1 cm^2^/week, 22(19.13%) were1–2 cm^2^/week and 38(33.04%) were > 2 cm^2^/week whereas, the overall mean time to healing was 42 ± 5.592 days.

From the total diabetes foot ulcer patients, a total of 77(67%) of ulcers were progressed to infection and 38(33%) of them did not. From the patients who developed an infection, gram- positive organisms were identified in 42(54.55%), gram-negative were identified in 20(25.97%) and polymicrobial were seen in 15(19.48%) [16].

From the total patient’s given antibiotics, a total of 38(49.35%) of them were prescribed appropriately and 39(50.65%) were prescribed inappropriately whereas, 38 (49.35%) of the diabetic foot ulcer patients were never given antibiotics. Individual antibiotics prescribed includes; cloxacillin 56 (34.15%), metronidazole 43 (26.22%), ceftriaxone 33 (20.12%), ampicillin 9(5.49%), chloramphenicol 8(4.88%), gentamycin 5 (3.05%),ceftazidime 4(2.44%), ciprofloxacin 3 (1.83%), vancomycin 2 (1.22%), and amoxicillin 1(0.61%).

Neither age nor gender had an association with the outcomes of foot ulcers whereas; advanced grade of ulcer and inappropriate antibiotics use had an association with the healing of diabetes foot ulcers. Patients prescribed with inappropriate antibiotics were 2 times more likely to be amputated as compared to the patients given appropriate antibiotics (AHR = 2.14;95%CI:1.64,10.63). The higher grades of foot ulcers, the worse the outcome of healing. Diabetes foot ulcer patients presented with grade ≥ 4 were 1.6 times more likely to be amputated as compared to the patients having grade < 4(AHR = 1.59;95%CI:1.49,7.48) (Table 2).

**Table 2:** Predictors of amputation rate of diabetes foot ulcer and associated factors in Nekemte referral hospital, west Ethiopia, 2018

Variables		Amputation		CHR(95% CI)	AHR(95% CI)	*P*- value
		Yes	No			
		N (%)	N (%)			
Sex	Male	19 (29.69)	45 (70.31)	1.46 (0.67–3.52)		0.158
	Female	16 (31.37)	35 (68.63)	1		
Age	18–27	2 (12.50)	14 (87.50)	1		0.115
	28–37	4 (28.57)	10 (71.43)	1.76 (0.79–3.75)		0.184
	38–47	7 (46.67)	8 (53.33)	2.10 (0.98–6.95)		0.150
	48–57	8 (33.33)	16 (66.67)	3.24 (0.69–7.74)		0.228
	58–67	9 (34.62)	17 (65.38)	2.76 (0.89–8.56)		0.187
	68–77	5 (25.00)	15 (75.00)	3.61 (0.94–7.76)		0.223
Types of DM	Type 2 DM	23 (37.70)	38 (62.30)	1.47 (0.86–6.73)		0.074
	Type 1 DM	12 (22.22)	42 (77.78)	1		
Residence	Rural	17 (29.82)	40 (70.18)	2.73 (0.48–7.73)		0.247
	Urban	18 (31.03)	40 (68.97)	1		
Co-morbidity	Yes	23 (39.66)	35 (60.34)	2.74 (0.70–7.47)		0.190
	No	12 (21.05)	45((78.95)	1		
Wagner grades	Grade < 4	19 (21.84)	64 (78.16)	1	1	0.005*
	Grade ≥ 4	16 (57.14)	16 (20.00)	4.70 (1.96–8.63)	1.59 (1.49–7.48)	
Antibiotics given	Appropriate	14 (36.84)	24 (63.16)	1	1	0.017*
	Inappropriate	21 (53.85)	18 (46.15)	3.48 (1.84–9.53)	2.14 (1.64–10.63)	

^*^Shows statistically significant *p*-value < 0.05at 95% CI

**Fig. 1:**
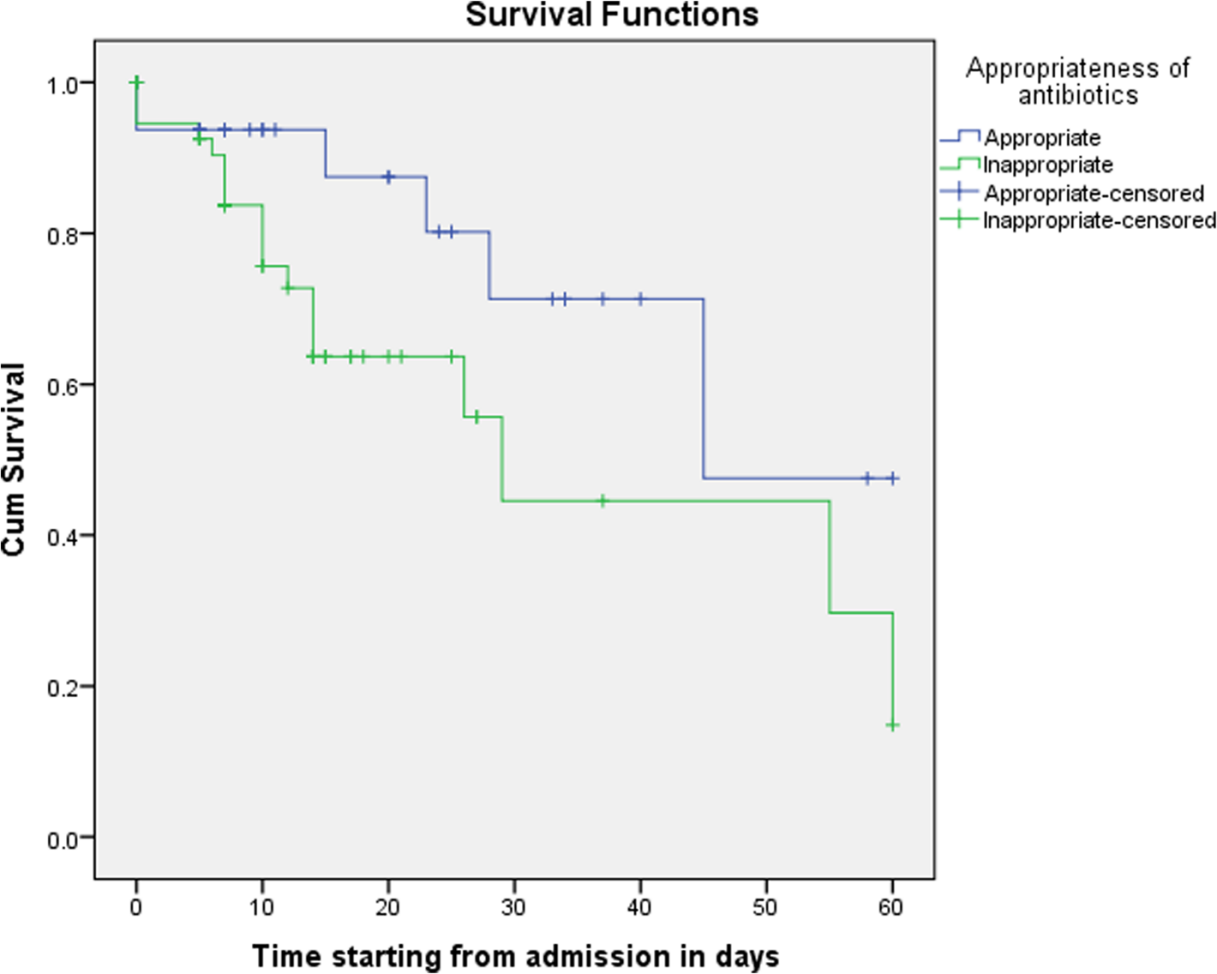
Kaplan–Meier curves for amputation-free survival of diabetes foot ulcer patients over 60 days, depending on the appropriateness of antibiotics prescribed

The Kaplan–Meier survival analyses of the patients who were given appropriate and inappropriate antibiotics showed that the healing time of diabetes foot ulcer patients who were given inappropriate antibiotics were prolonged than diabetes foot ulcer patients who were given appropriate antibiotics (Fig. 1).

**Fig. 2:**
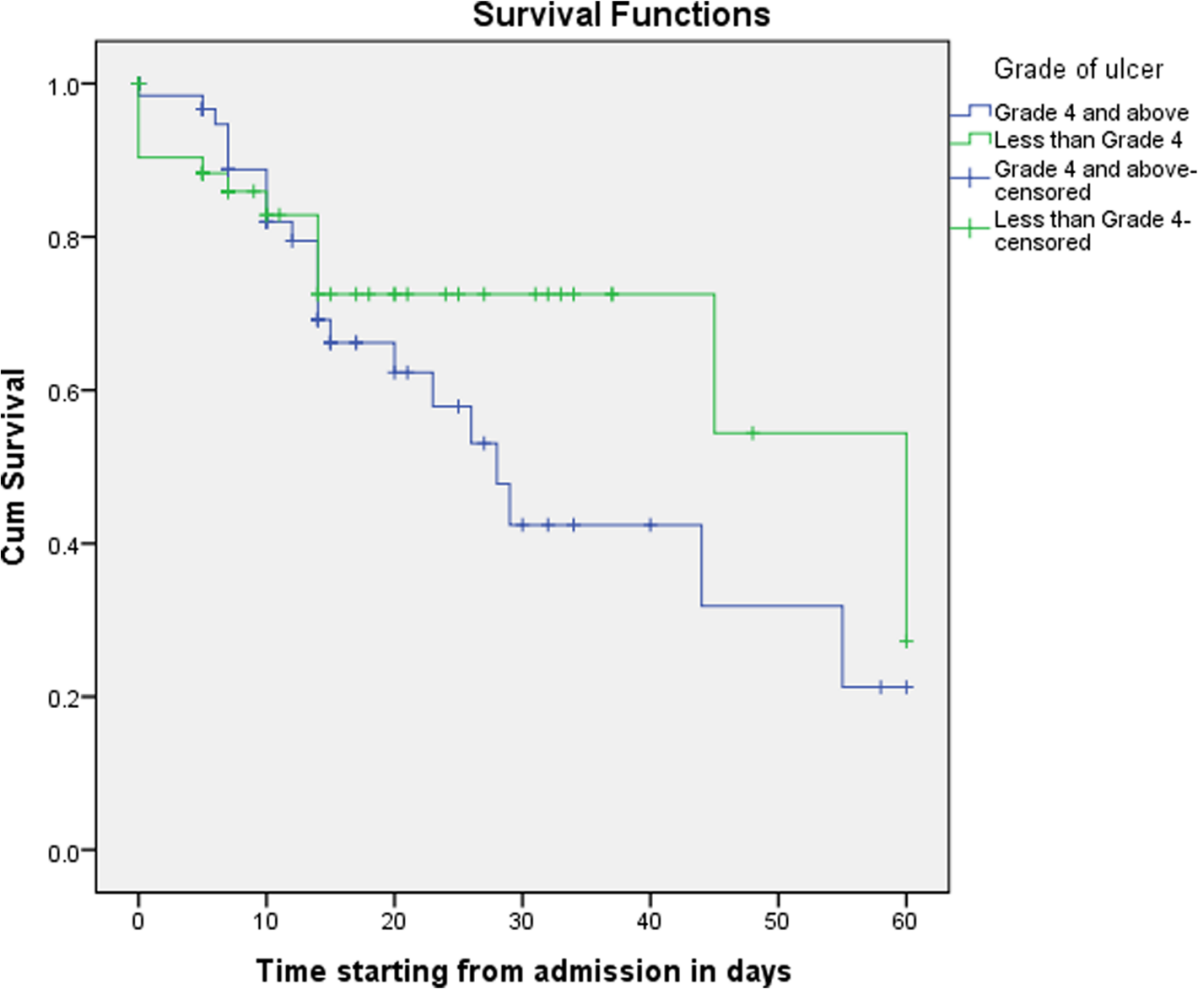
Kaplan–Meier curves for amputation-free survival of diabetes foot ulcer patients over 60 days, depending on the grades of diabetic foot ulcers

The Kaplan–Meier survival analyses of the patients who were on Wagner grade less than 4 and grade 4 and above showed that the healing time of diabetes foot ulcer patients who were on the advanced stage of diabetes foot ulcer was prolonged than diabetes foot ulcer patients who were at an earlier stage (Fig. 2).

## Discussion

The study was aimed to identify the amputation rate of diabetes foot ulcer and its predictors. Appropriate anti-microbial therapy is essential for diabetes foot ulcers that progressed to infection, unlike this; prescribing antibiotics for uninfected foot ulcers can results in unnecessary therapy, increase cost, and risk of antibiotic resistance [17]. Therefore, appropriate use of antibiotics is a very crucial problem for clinicians. Duration of antibiotic therapy for a diabetes foot infection should be based on the severity of the infection and clinical response to therapy, and antibiotic therapy can generally be discontinued when the patients don’t show any clinical presentation of infection [13]. One medical doctor, one nurse were selected as data collectors as they had experience in treatment of DFU.

The study conducted by Fatma I. Abo El-Ela et al. showed appropriate treatment of diabetes foot infection by amoxicillin and doxycycline help to prevent bacterial growth and ulcers that heal the wounds within a short period [18]. According to the study done in China by Chu et al., for moderate/severe infection, the healing rate was rapid in patients given appropriate antibiotic therapy [17]. This is similar to our study in which appropriate antibiotics prescription can fasten the healing rate of diabetes foot ulcers. This is because in China and the Nekemte referral hospital the antibiotics were started when the patients reach the advanced stage of grade. In our study, almost half of the antibiotics were prescribed inappropriately due to a lack of clinical pharmacy service that can improve the rational antibiotics prescription. Therefore, because of excessive and inappropriate use of antibiotics for treating diabetes foot infections, resistance to the usually employed bacteria wills possibly increasing to alarming levels in the study area unless tackled. The International Working Group on the Diabetic Foot Also recommends selecting antibiotic agents for treating diabetic foot infection from among those that have demonstrated efficacy for diabetic foot infection in clinical studies [19].

On the contrary, the study conducted in Denmark revealed that the antibiotics given were not significantly associated with the healing rate of diabetes foot ulcers [20]. This was in agreement with our study.

In our study, the rate of healing of almost half of the patients’ wounds was very rapidly, i.e., greater than 2 cm^2^/week. On the contrary, in Saudi Arabia the rate of healing of more than three-fourths of patients’ wounds was very slow, i.e., less than 1 cm2/week [1]. The rapid healing rate of diabetes foot ulcers in our study was due to initial Wagner’s grades of the majority of diabetes foot ulcer patients.

We found the average duration for ulcer healing in our study was 42 days, which was a relatively short time compared to the study of Saudi Arabia of 3 months, Denmark of about 6 months and the USA of 133 days [10, 20, 21], and in the UK the healing rate was 2 months which was almost similar to our study [22]. The accelerated healing times might be due to the superficial infections of foot ulcer in majority of cases in our study.

The finding was not similar to those of Markuson et al., who identified a difference with in the type of diabetes and healing time. More patients in type one diabetes mellitus had rapid healing rate than type two diabetes mellitus [21]. In UK the healing rate was similar in both type one and two diabetes mellitus patients [22]. This is similar to our study in which type of diabetes couldn’t affect the healing rate.

Advanced Wagner grades (≥ grade 4) predicted the amputation rate of diabetes foot ulcers in our study. This is because gangrene was developed at an advanced grade of foot ulcer which finally results in amputation. Similarly, Sung Hun Won, et al. showed that advanced grade of ulcer had a risk of amputation [23], similar to our study. This is due to the patients at a higher levels of Wagner’s grade seek hospital after gangrene was developed in our study.

The amputation rates of foot ulcers were higher than the study done by Morbach et al. [24]. This is because all amputation levels (minor and major) were included in our study over the study period of 3 months. The study conducted in Brazil revealed that about 11.7% of the diabetic foot ulcer patients were undergone amputation [25], which was lower than our study. This may be due to a lack of quality of health service, inadequate healthcare professionals to provide care for the patients in our study. The healing rate was comparable with the study conducted by Akturk A, etal, in which 67% of the DFU were healed [26].

### Strength and limitation of the study

As strength, the study was conducted among DFU patients as the foot complication of diabetes mellitus patients is increasing in the developing world and may be used as baseline information for other researchers. As limitations, culture, and sensitivity tests were not done to identify the specific strains of the pathogen. Further the follow-up period was short, thus failing to take into account any non- healing ulcers after 3 months and patients were followed by telephone not by face to face interviews. Despite the mortality was not reported in our study, further research should be conducted to identify the mortality rate. Lastly, there might be selection bias due to convenience sampling technique of the study.

## Conclusion

The healing rate of diabetes foot ulcers was rapid and the majority of them were healed within 2 months. The amputation rate of diabetes foot ulcers was rapid for patients prescribed inappropriate antibiotics therapy and an advanced grade of foot ulcers. Therefore, the presence of clinical pharmacists plays a pivotal role to facilitate and promote the appropriate use of antibiotics by intervening different problems in prescribing, dispensing, and providing necessary advice for the patients, and health care professionals regarding the overall issues related to drugs. Besides the daily care, special attention should be given for patients having advanced grades of diabetes foot ulcers.

## Data Availability

The datasets used during the study are available from the corresponding author on a reasonable request.

## Abbreviations

AHR: Adjusted hazard ratio
BMI: Body mass index
CHR: Crude hazard ratio
CI: Confidence interval
DFU: Diabetic foot ulcer
DM: Diabetes mellitus
IDSA: Infectious Diseases Society of America
IWGDF: International Working Group on the Diabetic Foot
LEA: Lower extremity amputation
NRH: Nekemte referral hospital
SD: Standard deviation
SPSS: Statistical package for social sciences
UK: United Kingdom
